# Congenital presentation of synchronous Atypical Teratoid Rhabdoid Tumor and Malignant Rhabdoid Tumor of the urinary bladder in a term infant

**DOI:** 10.4322/acr.2020.205

**Published:** 2020-11-20

**Authors:** Vivian Tang, Peter Michael Conner, Jason Paul Tovar, Regina Frances Gandour-Edwards, Reuben Antony, Matthew Bobinski, Michael Steven Brent Edwards, Mirna Lechpammer

**Affiliations:** 1 University of California Davis, Department of Pathology and Laboratory Medicine, Sacramento, CA, USA; 2 Sacramento County Coroner’s Office. Sacramento, CA, USA; 3 University of California Davis, Sacramento, Department of Pediatrics. Sacramento, CA, USA; 4 University of California Davis, Sacramento, Department of Radiology. Sacramento, CA, USA; 5 University of California Davis, Sacramento, Department of Neurosurgery. Sacramento, CA, USA

**Keywords:** Rhabdoid Tumor, Brain Neoplasms, Urinary Bladder Neoplasms, Autopsy, Infant

## Abstract

Atypical teratoid/rhabdoid tumor (AT/RT) is a rare central nervous system (CNS) tumor diagnosed primarily in infants and usually portends a poor prognosis. Despite being the most common embryonal tumor in children less than 1 year old, diagnosis is difficult to make based on clinical findings or imaging alone. A complete diagnosis of AT/RT requires identification of loss of integrase interactor 1 (INI1) protein or the SWI/SNF-related, matrix-associated, actin-dependent regulator of chromatin, subfamily b, member 1 (*SMARCB1*) gene, in its most common presentation. Moreover, their presentation with other primary rhabdoid tumors in the body raises significant suspicion for rhabdoid tumor predisposition syndrome (RTPS). We report a case of a one-month-old infant admitted for worsening emesis and failure to thrive, who was later found to have brain and bladder masses on radiologic imaging. Autopsy with subsequent immunoprofile and molecular testing were crucial in establishing the absence of INI1 nuclear expression and possible homozygous deletion of *SMARCB1* in the urinary bladder tumor tissue. Sequencing of the peripheral blood demonstrated probable single copy loss at the *SMARCB1* locus. The constellation of findings in tumor and peripheral blood sequencing suggested the possibility of germline single copy *SMARCB1* loss, followed by somatic loss of the remaining *SMARCB1* allele due to copy neutral loss-of-heterozygosity. Such a sequence of genetic events has been described in malignant rhabdoid tumors (MRT). Dedicated germline testing of this patient’s family members could yield answers as to whether rhabdoid tumor predisposition syndrome will continue to have implications for the patient’s family.

## CASE REPORT

A 41-day-old female infant born at gestational age 39 weeks and 1 day to a 38-year-old G5T1P1A3L1 mother presented with worsening non-bloody, non-biliary emesis and fatigue since approximately two weeks of life. Maternal gestational history had been notable for a previous intrauterine fetal demise at 33 gestational weeks, for which fetal autopsy and genetic workup at an outside hospital were negative. She subsequently had three spontaneous first-trimester abortions and had undergone in-vitro fertilization (IVF) therapy for the past five years. Family history is notable for the carrier status of genetic syndromes in the mother and father of the infant, revealed by genetic workup at an outside IVF clinic. The infant’s mother is homozygous for a methylenetetrahydrofolate reductase (*MTHFR*) C677T mutation with normal homocysteine levels detected on her last prenatal visit and is also a carrier for peroxisomal biogenesis factor 1 (*PEX1*) related Zellweger syndrome. The infant’s maternal great grandfather was reported to have died of unspecified brain cancer at age 35. On the paternal lineage, the infant’s father is a heterozygote carrier for Gaucher disease, and her paternal great-great-grandfather was reported to have had a history of unspecified gastrointestinal tumors. Both parents are of Caucasian descent.

This patient was conceived spontaneously, but the pregnancy was complicated by the development of vanishing twin syndrome after an initial dichorionic-diamniotic pregnancy. Delivery of the infant was via normal spontaneous vaginal delivery with APGAR scores of 8 and 9 at 1 and 5 minutes of life.

The infant was reported to have had normal behavior, feeding, and stooling until day 10 of life. At this point, she began experiencing colic and vomiting with feeds necessitating frequent positioning. At her 2-week check-up, she had not regained her birth weight. This, combined with worsening emesis and fatigue, prompted her family to present to the Emergency Department, where she was admitted for failure to thrive.

**Figure 1:**
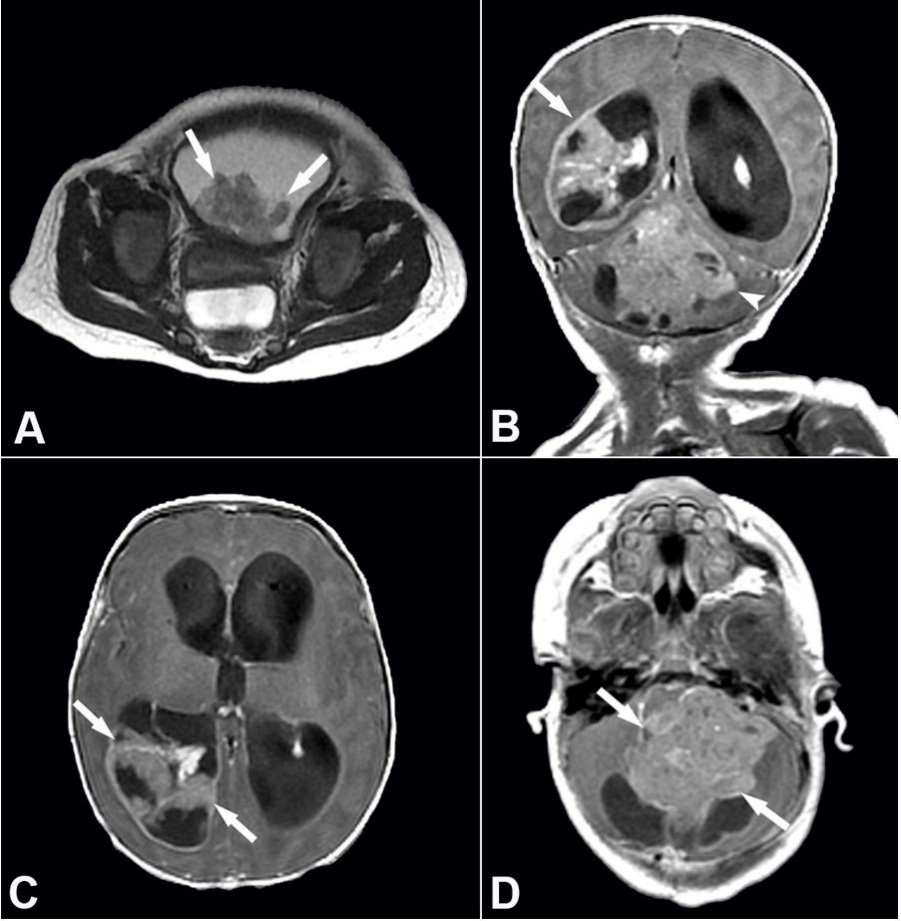
Radiologic imaging of urinary bladder and brain masses. **A –** Axial T2-weighted image of the pelvis shows a large lobulated mass in the dependent portion of the urinary bladder (arrows); **B –** Coronal T1-weighted contrast-enhanced image of the brain demonstrates a large heterogeneous mass in the right lateral ventricle (arrow) and the fourth ventricle (arrowhead); Axial T1-weighted contrast-enhanced images of the brain demonstrate a large heterogeneous mass (arrows) in **C –** the right lateral ventricle and **D –** the fourth ventricle.

On admission, initial abdominal ultrasound ruled out pyloric stenosis or gastrointestinal obstruction. Because the patient’s urine culture resulted positive for E. coli, antibiotics were initiated, and a pelvic ultrasound was performed. This revealed a 2 cm urinary bladder mass, which subsequent pelvic magnetic resonance imaging (MRI) was characterized as a nodular/polypoid mass-like lesion rising off the posterior inferior wall of the bladder (Figure 1A). Cystoscopy and transurethral resection and biopsy of the bladder mass were performed on day 6 of admission. A preliminary diagnosis of embryonal rhabdomyosarcoma was provided at the time, pending additional immunostains.

In light of the patient’s ongoing emesis, a head ultrasound was also performed on day 6, which demonstrated massive hydrocephalus and a large hyperechoic lesion centered in the right lateral ventricle. A follow-up brain and spine MRI was performed, and this demonstrated a lobulated heterogeneous enhancing mass centered in the fourth ventricle and invading both cerebellar hemispheres (Figures 1B and D), as well an additional mass in the right occipital horn extending into the atrium (Figures 1B and C); evidence of diffuse leptomeningeal dissemination was noted on the contrast MRI sequences. The differential diagnoses at this stage included atypical teratoid/rhabdoid tumor (AT/RT), medulloblastoma, choroid plexus carcinoma, or anaplastic ependymoma. Macrocephaly was not noted on imaging or clinical record at the time of diagnosis. Due to the extensive growth and degree of invasion, the prognosis was deemed very poor. After consulting with a multi-disciplinary team, the decision was made to pursue comfort care, and on day 7 of admission, the patient was discharged to a home hospice where she died two days later.

## AUTOPSY FINDINGS

The decedent weighed 3500 g (reference range [RR]; 2800-4100 g),^1^ with a crown-heel length of 55 cm (mean ± SD; 55.8 ± 3.3 cm)^2^ and a crown-rump length of 40 cm (mean ± SD; 39.3 ± 2.0 cm).^2^ Head circumference measured 38.1 cm (RR; 35.5-40.5 cm).^1^ Facial features appeared normally developed and unremarkable, as were the neck, external genitalia, anus, and extremities. Both hands were contracted in a grasping manner.

**Figure 2:**
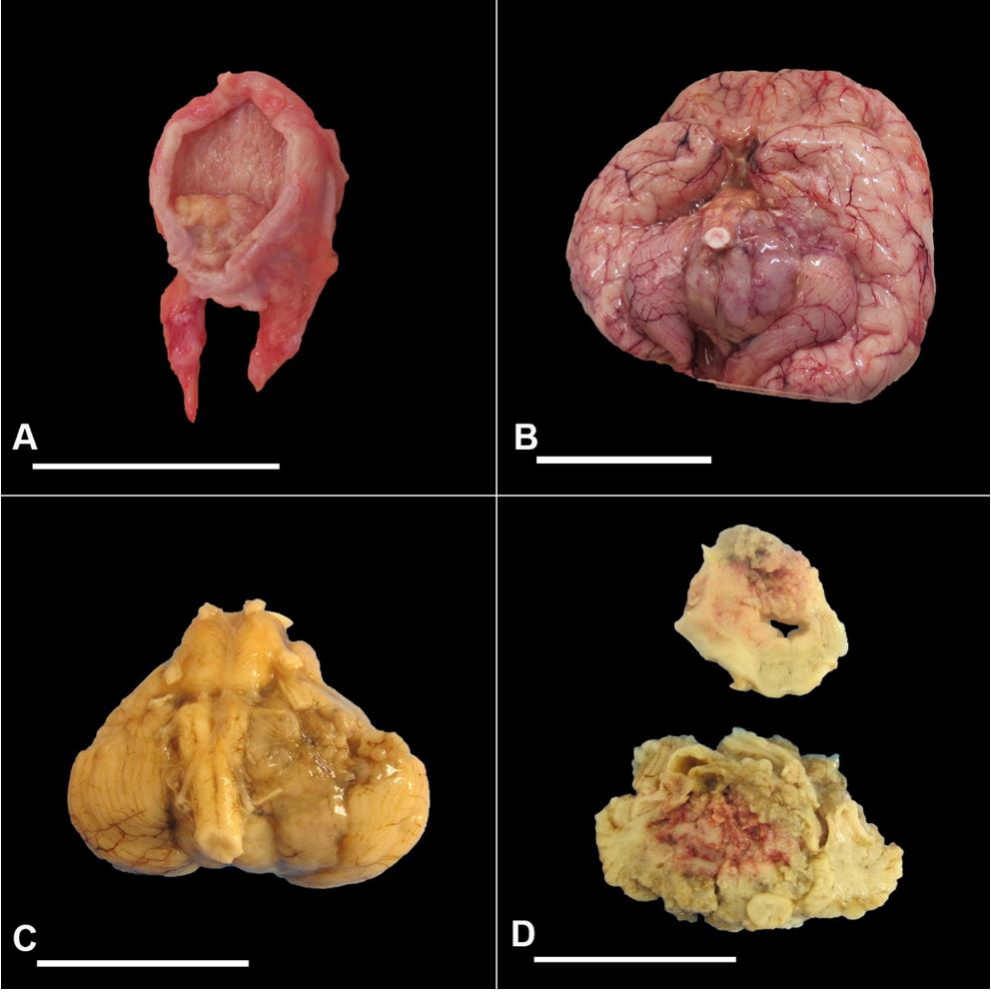
Gross appearance of urinary bladder and brain masses. **A –** Exophytic mass can be seen on the right posterior-lateral-inferior wall of the bladder, measuring 2.0 × 1.5 × 1.0 cm; **B –** Brain tumor visible in the posterior fossa, with both the dorsal and ventral surfaces of the cerebellum appearing grossly necrotic; **C –** Cerebellum and brain stem separated from the cerebrum at the level of the rostral midbrain with tumor visible on the right; **D –** Transversely cut surfaces of the cerebellum and brain stem showing a pink necrotic tumor measuring 4.5 × 4.0 × 3.5 cm. Scale bars 5 cm.

**Figure 3:**
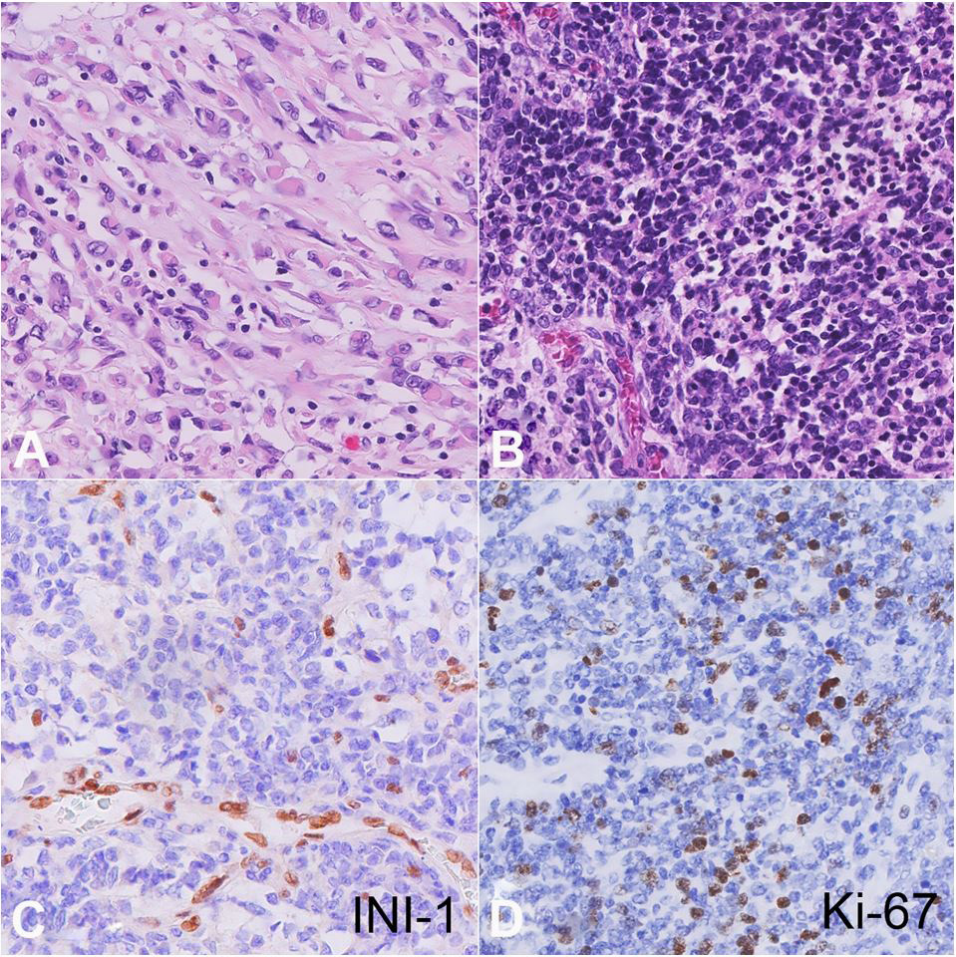
Histopathology of the urinary bladder and brain tumors. **A** – Bladder tumor consisting of pleomorphic round-to-spindle shaped cells focally embedded into loose myxoid stroma interspersed with densely packed areas of small rhabdoid cells containing round to oval nuclei with vesicular chromatin and prominent nucleoli (H&E, 400x); **B** – Histopathology of brain neoplasm shows primitive malignant blue cell neoplasm consisting of densely packed neoplastic cells growing in sheets with very few cells resembling rhabdoid morphology (H&E, 400x); **C** – Loss of INI-1 nuclear expression in brain tumor cells with positive staining in endothelial cells as an internal control (400x); **D** – Brain tumor cells showing increased and proliferative activity (Ki-67, 400x).

The autopsy was restricted to the bladder and brain only. Midline dissection revealed no visible abnormalities to the small bowel and colon. The ureters were patent and dilated, and the internal reproductive organs unremarkable except for enlargement of the bilateral ovaries with cystic changes. The urinary bladder appeared enlarged, but the exterior surface was grossly unremarkable, smooth, and tan-pink. Upon opening the bladder, a tan-pink, fungating, exophytic mass on the right posterior-lateral-inferior wall was revealed (Figure 2A), measuring 2.0 cm (medial-lateral) by 1.5 cm (superior-inferior) by 1.0 cm (anterior-posterior). Microscopic evaluation of the bladder tumor showed both hypocellular areas with a loose myxoid stroma and more densely packed areas with a small round to oval cells with vesicular chromatin, prominent nucleoli, and scant cytoplasm (Figure 3A). Other areas showed cells with rhabdoid features such as eccentric nuclei and eosinophilic cytoplasmic inclusions. Additionally, rare atypical mitotic figures were seen. The tumor demonstrated focal extension into the underlying muscularis propria. The uninvolved bladder showed benign squamous epithelium, and fibromuscular stroma with focal mononuclear cell infiltrates. The urethra and ureters were uninvolved by the tumor. Immunostains on the bladder mass were negative to Melan-A and Synaptophysin, excluding the possibility of melanocytic or neuroendocrine origin of this tumor, and showed loss of integrase interactor 1 (INI1) staining in the tumor nuclei. Based on immunoprofile and morphology, this appeared most consistent with malignant rhabdoid tumor (MRT), rather than originally suspected rhabdomyosarcoma.

Standard coronal incision and reflection of the scalp revealed no abnormalities in the calvarium. The brain had a fixed weight of 540 g (RR; 411-741 g).^3^ The cerebral hemispheres were notable for a deep left olfactory sulcus, thinning of the corpus callosum, and crowding of the occipital and frontal gyri. The brainstem was noted to have yellow discoloration, particularly in the midbrain and pons, likely attributed to necrosis and tumor involvement. The tumor was visible in the posterior fossa (more so on the left side), with both the dorsal and ventral surfaces of the cerebellum appearing grossly necrotic (Figure 2B). Uncal herniation was present. Cut surfaces of the cerebral cortex revealed a necrotic pink tumor involving the right hemisphere measuring 8.0 cm by 4.5 cm by 4.0 cm. It appeared that the tumor extended into the cortical surface, filled the right lateral ventricle, and showed a potential degree of midline extension. The component in the fourth ventricle resulted in severe obstructive hydrocephalus, and the components in the right atrium and occipital horn may have contributed to herniation and subsequent compression of the cerebral peduncle. The cerebellum and brain stem separated from the cerebrum at the rostral midbrain level weighed 70 g (Figure 2C). Transversely cut surfaces revealed a pink necrotic tumor 4.5 cm by 4.0 cm by 3.5 cm in size involving both cerebellar hemispheres, vermis, and brain stem (Figure 2D).

Microscopic examination of the brain tumor tissue revealed a primitive malignant blue cell neoplasm (Figure 3B) with extensive necrosis and leptomeningeal spread. Morphology of the tumor was heterogeneous, consisting of variably sized neoplastic cells predominantly growing in sheets. Some of the larger cells were oval-shaped with larger nuclei containing vesicular chromatin, prominent nucleoli, and scant eosinophilic cytoplasm; the other cells were smaller with more compact chromatin and higher nuclear-to-cytoplasmic (N:C) ratios. Unlike the urinary bladder tumor, neoplastic cells in the brain contained very few cells with rhabdoid morphology.

Microscopic examination of bilateral cerebellar lobes, vermis, rostral pons, and caudal medulla showed involvement with the same primitive malignant blue cell neoplasm and necrosis described above. The section of the medulla with involved tumor underwent immunostaining, and was notably negative for INI1 in the tumor cells (although positive in endothelial cells) (Figure 3C) and 5-7% (focally up to 20%) positive for Ki-67 (Figure 3D). There was additionally positive to weakly-positive expression for GFAP, Synaptophysin, and Neurofilament, as well as EMA positivity in a subpopulation of rhabdoid cells. Based on histology and immunophenotype, the neuropathological diagnosis was determined to be AT/RT.

Taken together, the findings of the autopsy suggest the cause of death being obstructive hydrocephalus from the tumor as well as extensive destruction of the cerebral cortex, cerebellum, and brain stem secondary to tumor invasion, leading to herniation and loss of function of vital respiratory and cardiovascular control centers.

After completion of the autopsy, samples of peripheral blood and bladder tumor tissue were submitted to the University of California San Francisco (UCSF) Clinical Cancer Genomics Laboratory for molecular testing. The UCSF500 Gene Panel (University of California, San Francisco, San Francisco, California) was performed using capture-based next-generation sequencing, which targeted and analyzed the coding regions of 479 cancer genes and select introns of 47 genes. The results indicated deep loss (i.e., possible homozygous deletion) of SWI/SNF-related, matrix-associated, actin-dependent regulator of chromatin, subfamily B, member 1 (*SMARCB1*) in the urinary bladder tumor tissue. Sequencing of the peripheral blood demonstrated probable single copy loss at the *SMARCB1* locus. Furthermore, tumor sequencing demonstrated somatic copy neutral loss-of-heterozygosity of most or all chromosome 22, the chromosome on which *SMARCB1* is expressed. This constellation of findings suggested the possibility of germline single copy *SMARCB1* loss, followed by somatic loss of the remaining *SMARCB1* allele due to copy neutral loss-of-heterozygosity. Such a sequence of genetic events has been described in MRT.

## DISCUSSION

This case report describes an infant with a presentation of worsening emesis and a rapid decline in the context of MRT of the bladder and AT/RT of the brain.

This patient was first diagnosed with a bladder mass on hospital admission, which was identified on autopsy as MRT of the bladder. The origin of MRTs, as a class, first arose with a description of a Wilms tumor with a distinct rhabdomyosarcomatoid subtype;^4^ this entity later became known to be the rhabdoid tumor of the kidney (RTK). Since then, primary MRTs have been found in various anatomic locations, including soft tissue^5^ and the CNS.^6^ Cases of MRT in the bladder have been rarer, nonetheless, and alterations in *SMARCB1*/INI1 are usually implicated in such cases.^7^

The patient was also found to have a synchronous malignant neoplasm of the brain, subsequently diagnosed as an AT/RT. Epidemiologically, AT/RT is a rare type of brain tumor, representing approximately 1-2% of all pediatric brain tumors.^8^ However, because it is the most common embryonal tumor in children less than 1 year old and greater than 80% of cases are diagnosed in children younger than 3 years old,^8^ it is estimated to represent 10% or more of all infant CNS tumors.^9^ Clinical features vary based on patient demographics and distribution of the tumor (namely, 21.8% of tumors occur supratentorially for infants 0–1 year, versus 69.0% for adolescents 6–18 years),^8^ but often include non-specific symptoms of lethargy, vomiting, and failure to thrive, as was the case in this infant. Prognosis is rather poor, with historical overall survivals at 6 months, 1 year, and 5 years of 65.0%, 46.8%, and 28.3%, respectively.^8^ However, a recently published report on the efficacy of high-dose chemotherapy and three-dimensional conformal radiation from the Children's Oncology Group trial ACNS0333 has dramatically improved event-free survival by 57% in patients < 36 months of age, compared to historical controls.^10^

Importantly, the genetic alteration most frequently associated with AT/RT and MRT is the loss of *SMARCB1* locus at chromosome 22q11.2.^11^ ^,^ ^12^ This patient was found to have a loss of expression of *SMARCB1* histologically, as seen by both brain and bladder tissues staining negative for INI1 (*SMARCB1* protein) in tumor cell nuclei. This was significant because the immunohistochemical absence of INI1 is both a sensitive and specific diagnostic test for AT/RT.^13^ Loss of *SMARCB1* was further supported by next-generation sequencing of peripheral blood and bladder tumor tissue, where the results showed the bladder tumor having a deep (i.e., possible homozygous) deletion of *SMARCB1* and the single-copy loss at the *SMARCB1* locus detected in the peripheral blood. Although the differential diagnosis for tumors with deficiency of *SMARCB1*/INI1 is wide and includes entities such as epithelioid malignant peripheral nerve sheath tumor, extraskeletal myxoid chondrosarcoma, renal rhabdoid tumor, and epithelioid sarcoma,^14^ the clinical and histopathological presentation, in this case, most closely resembles AT/RT.

This highly unusual simultaneous presentation of two rare rhabdoid neoplasms, combined with a maternal gestational history of recurrent pregnancy losses and various genetic syndromes in the family history raise concern that this patient likely has rhabdoid tumor predisposition syndrome (RTPS). RTPS is characterized by a propensity of developing MRTs due to the loss of *SMARCB1*, or more rarely, *SMARCA4* expression.^13^ It is worthwhile pursuing molecular genetic workup in suspected cases, because based on epidemiology alone, an estimated 25-35% of newly diagnosed patients with rhabdoid tumors will have a germline pathogenic variant in *SMARCB1*.^15^ ^-^ ^17^ After all, this patient’s *SMARCB1* deletion was confirmed on postmortem tissue, and there was an early onset of rhabdoid tumors, advanced stage of tumors at diagnosis, and synchronous tumors, several of the criteria for RTPS were met.^15^ Furthermore, even though there is no known family history of rhabdoid tumors in this patient and present literature search reveals no clear associations with MTHFR mutation, Gaucher disease, or Zellweger syndrome, a potential correlation could exist with the unspecified brain tumor on the maternal lineage. Hence, the identification of a germline pathogenic variant in *SMARCB1* through molecular genetic testing could have meaningful implications for future decisions regarding childbearing for this family, as well as for the management of potential manifestations in relatives with appropriate surveillance. However, at this time, the parents have not chosen to undergo predictive genetic testing for hereditary RTPS. The autopsy nonetheless proved valuable in providing important clinicopathological information. Although the autopsy was restricted to the brain and bladder only, and a full autopsy may have contributed to assessing for additional malignancy (e.g., the kidneys), the full extent of the diagnosis could not have been appreciated with clinical findings alone. The knowledge gained through autopsy could contribute to a fuller understanding of this devastating condition.

## CONCLUSION

MRT of the bladder and AT/RT of the brain are independently rare entities with very poor prognoses. When observed simultaneously, as in this case of a 41-day-old infant with a rapid clinical decline, the prognosis is devastating, and significant concern arises for the presence of RTPS. Whereas most MRT cases are found in the kidney and CNS, this is the first reported case, to our knowledge, of INI1-negative AT/RT presenting with MRT of the bladder. The autopsy was valuable in that the diagnosis was augmented by the ability to sample tissue from both regions, and the molecular genetic workup affirming the presence of *SMARCB1* deletion shed light onto a presentation of this syndrome not previously characterized.
